# Auditory and Cognitive Deficits Associated with Acquired Amusia after Stroke: A Magnetoencephalography and Neuropsychological Follow-Up Study

**DOI:** 10.1371/journal.pone.0015157

**Published:** 2010-12-02

**Authors:** Teppo Särkämö, Mari Tervaniemi, Seppo Soinila, Taina Autti, Heli M. Silvennoinen, Matti Laine, Marja Hietanen, Elina Pihko

**Affiliations:** 1 Cognitive Brain Research Unit, Institute of Behavioural Sciences, University of Helsinki, Helsinki, Finland; 2 Finnish Centre of Excellence in Interdisciplinary Music Research, University of Jyväskylä, Jyväskylä, Finland; 3 Department of Psychology, University of Jyväskylä, Jyväskylä, Finland; 4 Department of Neurology, Helsinki University Central Hospital, Helsinki, Finland; 5 Department of Radiology, Helsinki University Central Hospital, Helsinki, Finland; 6 Department of Psychology, Åbo Akademi University, Turku, Finland; 7 BioMag Laboratory, Hospital District of Helsinki and Uusimaa, HUSLAB, Helsinki University Central Hospital, Helsinki, Finland; 8 Brain Research Unit, Low Temperature Laboratory, Aalto University School of Science and Technology, Espoo, Finland; Johns Hopkins Institute for Cell Engineering, United States of America

## Abstract

Acquired amusia is a common disorder after damage to the middle cerebral artery (MCA) territory. However, its neurocognitive mechanisms, especially the relative contribution of perceptual and cognitive factors, are still unclear. We studied cognitive and auditory processing in the amusic brain by performing neuropsychological testing as well as magnetoencephalography (MEG) measurements of frequency and duration discrimination using magnetic mismatch negativity (MMNm) recordings. Fifty-three patients with a left (n = 24) or right (n = 29) hemisphere MCA stroke (MRI verified) were investigated 1 week, 3 months, and 6 months after the stroke. Amusia was evaluated using the Montreal Battery of Evaluation of Amusia (MBEA). We found that amusia caused by right hemisphere damage (RHD), especially to temporal and frontal areas, was more severe than amusia caused by left hemisphere damage (LHD). Furthermore, the severity of amusia was found to correlate with weaker frequency MMNm responses only in amusic RHD patients. Additionally, within the RHD subgroup, the amusic patients who had damage to the auditory cortex (AC) showed worse recovery on the MBEA as well as weaker MMNm responses throughout the 6-month follow-up than the non-amusic patients or the amusic patients without AC damage. Furthermore, the amusic patients both with and without AC damage performed worse than the non-amusic patients on tests of working memory, attention, and cognitive flexibility. These findings suggest domain-general cognitive deficits to be the primary mechanism underlying amusia without AC damage whereas amusia with AC damage is associated with both auditory and cognitive deficits.

## Introduction

Abnormal brain development or brain damage can lead to a deficit in perceiving music, a condition known as amusia. Although studies of both congenital and acquired forms of amusia have surged during the past 20 years (for a recent review, see [1]), the neural mechanisms as well as the cognitive consequences associated with the condition are still unclear. Converging evidence from lesion studies [2] and modern structural MRI studies in individuals with congenital amusia [3]–[5] points to a network of temporal and frontal lobe areas, especially in the right hemisphere, as the critical brain substrate of amusia. However, the relative contribution of perceptual and cognitive factors to amusia is still under debate. Do amusic persons have difficulty already in discriminating low-level acoustical features, such as sound frequency and duration, that are crucial to music or is the deficit more related to an impaired cognitive analysis of music information?

Based on observed double dissociations between acquired amusia and impairment in the perception of speech (aphasia) and other non-musical sounds (auditory agnosia) (e.g. [6]–[8]), it has been proposed that there are mental modules in the brain that are specific to music perception [9]. However, recent behavioural evidence from congenital amusia suggests that amusic people can also have deficits in basic auditory discrimination [10], pitch memory [11]–[13], phonological and phonemic awareness [14], speech intonation processing [15]–[18], emotional prosody perception [19], and spatial processing ([20] but see also [21] for discrepant results). These findings suggest that the behavioural impairment in amusia may not be entirely specific to music perception. This view is also supported by evidence from studies of healthy subjects showing that music listening evokes widespread activation of many frontal, temporal, parietal, and subcortical areas related to for example attention, working memory, semantic and episodic memory, and emotional processing rather than being an endeavour of the auditory cortices alone (e.g. [22]–[25]).

Previously, the neural ability of amusic individuals to process music information has been studied with electroencephalography (EEG) by recording event-related potentials (ERPs) to acoustic changes within tone sequences or melodies. Especially the mismatch negativity (MMN) component is well-suited for this purpose because it is an early ERP response elicited preattentively to any acoustical change in a repetitive sound stream [26], [27]. Current ERP evidence indicates that individuals with congenital amusia have relatively normal early responses (N2, MMN) but abnormal later attention-modulated responses (P3, P600) to small pitch changes within tone sequences or melodies ([28]–[30] but see also [31] for conflicting results). Also in a recent functional magnetic resonance imaging (fMRI) study, individuals with congenital amusia showed reduced activity in the right inferior frontal gyrus (IFG) to small pitch changes whereas the activity in their left and right auditory cortex (AC) was comparable to control subjects [32]. This is in line with the fMRI studies of healthy subjects showing that judging or making decisions about auditory stimuli recruits domain-general frontal areas [33], [34], such as the inferior frontal gyrus (IFG), which are involved in processing response conflict, perceptual difficulty, novelty, and working memory [35]. Collectively, these results suggest that domain-general auditory and cognitive processes, mediated by neural structures beyond the AC, are linked to the music perception deficit in congenital amusia. However, very little is currently known about the contribution of auditory and cognitive deficits in acquired amusia.

Previously, there have been only two relatively small auditory ERP studies of acquired amusia (both with 12 patients). Münte and colleagues reported that stroke patients with acquired amusia had grossly reduced MMN responses to pitch changes [36] as well as decreased P3a responses to novel environmental sounds [37] compared with non-amusic patients and healthy control subjects. Interestingly, the amusic patients also showed worse performance on a behavioural auditory alertness test [37]. Although based on a relatively small number of patients, these results suggest that deficits in automatic sound-change detection and attention-orienting could be associated with acquired amusia. However, to date no study has systematically and directly explored which brain areas underlie acquired amusia and how it is related to other auditory and cognitive deficits.

In a previous study [38], we performed repeated neuropsychological testing in amusic and non-amusic patients (total n = 53) with a middle cerebral artery (MCA) stroke during a 6-month post-stroke period. We found that acquired amusia and its recovery were associated with a wide range of cognitive functions, especially attention, executive functioning, and working memory [38]. By using the same patient sample, the aim of the present magnetoencephalography (MEG) study was to explore whether the amusic and the non-amusic patients would differ in auditory discrimination, as indicated by the cortically generated magnetic mismatch negativity (MMNm) response to changes in basic acoustic features, such as sound pitch and duration, during recovery. Previous studies using almost identical stimuli have verified that healthy subjects are easily able to discriminate the changes in pitch (500 to 575 Hz) and duration (75 to 25 ms) used in the present study and also show robust MMN (MMNm) responses to these changes [39], [40]. Moreover, we sought to determine how the laterality of the cerebral damage as well as the presence of AC damage would influence the recovery of music perception and auditory discrimination in amusia. Specifically, we hypothesized that amusic patients with and without AC damage would show different patterns of MMNm and cognitive deficits (especially in tests of attention, executive functioning, and working memory) compared with non-amusic patients during the 6-month recovery period.

## Methods

### Subjects and procedure

Subjects (n = 53) were non-musician stroke patients recruited from the Department of Neurology of the Helsinki University Central Hospital (HUCH) to a randomized clinical trial about the effectiveness of music and audio book listening on stroke recovery (for a more detailed description of patient characteristics and methodology, see [41], [42]). All patients had an acute left (n = 24) or right (n = 29) MCA territory ischemic stroke verified by MRI, no prior neurological or psychiatric disease, drug or alcohol abuse and they were right-handed, ≤75 years old, Finnish-speaking, and able to co-operate. In addition, also patients who reported any problems in basic auditory perception (e.g., clearly worsened hearing, presbycusis, use of hearing aids, tinnitus, or Meniere's disease) before the stroke were excluded. As a part of the trial, all patients underwent a neuropsychological assessment of cognitive recovery and an MEG measurement of auditory discrimination 1 week, 3 months, and 6 months after the stroke as well as a structural 1.5 T MRI scan with routine sequences for stroke 2 weeks and 6 months post-stroke. The size and location of the lesion(s) were classified by neuroradiologists (authors T.A. and H.M.S) as previously described [41]. In addition, lesions of the auditory cortex were recorded. The study was approved by the Ethics Committee of the Hospital District of Helsinki and Uusimaa. The ethical permission granted the use of this data for both basic research related to auditory and music processing after stroke and for applied research related to the therapeutic effects of music listening. Since this study falls in the former category, it is clearly within the bounds of the ethical permission. All patients signed an informed consent stating that all information gathered from them during the study can be stored by the researchers according to the Finnish legislation on the concealment of confidential information and used anonymously for research purposes.

The patient sample and the data corpus in the present study is the same that was previously used to study the effects of music and audio book listening on stroke recovery [41], [42]. In those studies, music listening was found to enhance verbal memory and focused attention, prevent depressed and confused mood, and increase the amplitude of the frequency MMNm in the right hemisphere whereas audio book listening had no effect on cognition or mood but increased duration and frequency MMNm amplitudes in the right hemisphere [41], [42]. However, as we reported previously [38], the music, audio book, and control groups did not differ in the recovery of music perception. Moreover, the number of non-amusic and amusic patients was approximately the same in the music (10 vs. 8; *χ^2^* = 0.22, *p* = 0.637) and audio book (7 vs. 11; *χ^2^* = 0.89, *p* = 0.346) groups and their recovery did not differ in verbal memory and focused attention [38] or in the amplitude of the frequency MMNm [mixed-model ANOVA Time x Group interaction *F*(2, 30) = 0.20, *p* = 0.823 and *F*(2, 32)  = 0.19, *p* = 0.829, respectively] or the duration MMNm [*F*(2, 30)  = 1.09, *p* = 0.351 and *F*(2, 32)  = 0.42, *p* = 0.659, respectively] in the right hemisphere. Thus, we can conclude that comparing amusic and non-amusic patients using the MMNm and neuropsychological data from the three time points (1 week, 3 months, and 6 months post-stroke) was not in any way biased by the intervention.

### Assessment of cognition and music perception

Cognitive performance was assessed with an extensive (duration about 3 h) neuropsychological testing battery, which included tests of working memory, verbal learning and memory, verbal expression and comprehension, visuospatial cognition, executive functioning, and attention. Details concerning the neuropsychological tests are presented in Table 1. Parallel test versions of the memory tests were used in different testing occasions to minimize practice effects. Reaction time tests were always performed using the better, non-paretic hand. All assessments were carried out in a quiet room reserved for clinical neuropsychological assessments. The 1-week post-stroke assessment was carried out in two or three testing sessions to avoid interference due to fatigue. On the average, the assessments were spread over 2.98 days (range 2–7 days).

**Table 1:** Neuropsychological tests performed 1 week, 3 months, and 6 months post-stroke.

Test	Task of the subject	Reference
Working memory		
Digit span (WMS-R)	Recall number sequences	[43]
Memory interference	Recall sets of 3 words after interfering tasks	[44]
Verbal learning and memory		
Auditory list learning	Recall a list of 10 words (3 trials + delayed recall)	[44]
Story recall (RBMT)	Immediate and delayed recall of a narrated story	[45]
Verbal expression and comprehension		
Repetition (BDAE)	Repeat heard words and sentences	[46]
Reading (BDAE)	Read out words and sentences	[46]
Semantic fluency (CERAD)	Say words in the animal category in 60 s.	[47]
Naming (CERAD)	Name objects from line drawings	[47]
Short Token test	Comprehension of verbal instructions	[48]
Visuospatial cognition		
Clock task	Recognize time and draw clock hands	[44]
Copying designs	Draw copies of 4 geometric designs	[44]
Shortened BVRT	Draw 5 geometric designs from memory	[49]
Music cognition		
Shortened MBEA	Detect changes in musical melodies	[38], [50]
Executive functions and attention		
FAB	Perform a set of short mental and motor tasks	[51]
Phonemic fluency	Say words beginning with letter “s” in 60 s.	[44]
Balloons test	Cancel targets in a visuospatial array	[52]
Simple reaction time (CS)	Press key when visual target appears	[53]
Subtraction task (CS)	Press key after mental subtraction	[53]
Stroop task (CS)	Press key in a colour response conflict situation	[53]
Vigilance task (CS)	Press key when target letter appears (15 min)	[53]

Abbreviations: BDAE: Boston Diagnostic Aphasia Examination, BVRT: Benton Visual Retention Test, CERAD: The Consortium to Establish a Registry for Alzheimer's Disease, CS: CogniSpeed© reaction time software, FAB: Frontal Assessment Battery, MBEA: Montreal Battery of Evaluation of Amusia, RBMT: Rivermead Behavioral Memory Test, WMS-R: Wechsler Memory Scale - Revised.

As a part of the neuropsychological testing, also music perception was evaluated 1 week and 3 months post-stroke by using a shortened version [38] of the Montreal Battery of Evaluation of Amusia (MBEA) [50]. The original MBEA includes six subtests (each with 30 items) that measure different components of music cognition (scale, contour, interval, rhythm, and meter perception, and recognition memory). Using the same stimuli and structure as in the original MBEA, we created a shortened version, which included only 14 items per subtest and thereby reduced the length of the test from 1.5 h to 45 min. The use of a shorter version was crucial in the present study due to time constraints as well as patient fatigue and the severity of cognitive deficits in the acute post-stroke stage. The stimuli were presented using a portable CD player and head-arch headphones. Before the test, sound volume was adjusted to a clearly audible but comfortable level individually for each patient. Due to time constraints, we were unfortunately not able perform audiometry to verify the basic hearing ability of the patients. However, no particular auditory difficulties were observed during normal conversation or during the neuropsychological assessment.

**Figure 1:**
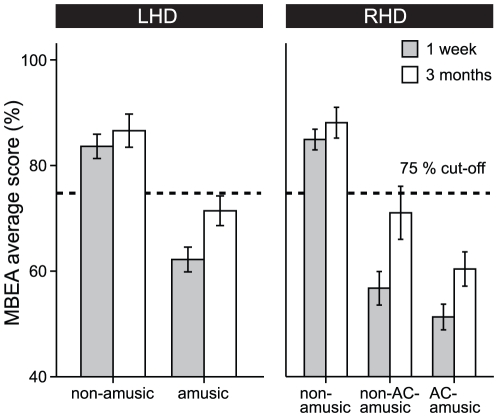
MBEA average scores of the patients 1 week and 3 months after the stroke. Data (mean ± SEM) are shown for non-amusic (n = 12) and amusic (n = 12) patients with left hemisphere damage (LHD) and for non-amusic (n = 9), non-AC-amusic (n = 9), and AC-amusic (n = 11) patients with right hemisphere damage (RHD). The dashed line indicates the amusia cut-off level (75%).

As it turned out, all of the 53 patients were able to perform the Scale and Rhythm subtests but only 44 were able to complete all the six subtests at the 1-week post-stroke stage. Since the Scale and Rhythm subtests were highly correlated with the other subtests and with each other [38], we opted to use their average score (referred to hereafter as the MBEA average score) in determining the presence of amusia in the sample of 53 patients. Based on the distribution of the MBEA average score (see [38] Figure 1) and the established cut-off values (2 SD below the normative mean) of the original MBEA [50], we classified the patients scoring less than 75% correct as amusic, resulting in 32 amusic and 21 non-amusic patients. Demographical and clinical characteristics of the patients are presented in [38] and in Table 2.

**Table 2:** Patient characteristics.

	Left hemisphere damage		*p* value	Right hemisphere damage			*p* value
	Amusic(n = 12)	Non-Amusic (n = 12)		AC-amusic (n = 11)	Non-AC-amusic (n = 9)	Non-amusic (n = 9)	
Gender (male/female) Age (years)	8/459.6 (8.6)	9/352.3 (8.5)	0.653 (*χ^2^*) 0.051 (*t*)	4/7	2/7	6/3	0.141 (*χ^2^*)
				61.2 (7.5)	59.7 (7.1)	61.2 (10.2)	0.857 (*F*)
Education (years)	9.3 (2.0)	13.8 (3.7)	0.002 (*t*)	9.5 (3.1)	10.4 (4.6)	12.1 (2.5)	0.277 (*F*)
Formal music training^a^	0 (0)	0.3 (0.9)	0.740 (*U*)	0 (0)	0 (0)	0 (0)	
Instrument playing^a^	0.7 (1.2)	1.9 (2.2)	0.198 (*U*)	1.4 (1.5)	0.8 (1.3)	1.8 (1.9)	0.476 (*K*)
Music listening prior to stroke^b^	3.2 (1.6)	4.1 (0.8)	0.198 (*U*)	2.9 (1.8)	4.1 (1.3)	3.7 (1.7)	0.403 (*K*)
Aphasia (no/yes)^c^	3/9	5/7	0.385 (*χ^2^*)	11/0	8/1	8/1	0.368 (*χ^2^*)
Visual neglect (no/yes)^d^	10/2	12/0	0.086 (*χ^2^*)	2/9	6/3	9/0	0.0001 (*χ^2^*)
Lesion size^e^	5.8 (2.2)	3.6 (1.6)	0.009 (*t*)	7.1 (1.4)	5.7 (2.6)	4.7 (3.0)	0.087 (*F*)
Frontal lesion (no/yes)	5/7	7/5	0.413 (*χ^2^*)	0/11	0/9	2/7	0.081 (*χ^2^*)
Temporal lesion (no/yes)	5/7	5/7		0/11	1/8	4/5	0.018 (*χ^2^*)
Auditory cortex lesion (no/yes)	9/3	11/1	0.264 (*χ^2^*)	0/11	9/0	9/0	<0.0001 (*χ^2^*)
Parietal lesion (no/yes)	4/8	5/7	0.673 (*χ^2^*)	1/10	6/3	6/3	0.006 (*χ^2^*)
Insular lesion (no/yes)	7/5	5/7	0.413 (*χ^2^*)	0/11	3/6	4/5	0.016 (*χ^2^*)
Subcortical lesion (no/yes)	9/3	6/6	0.203 (*χ^2^*)	2/9	5/4	4/5	0.189 (*χ^2^*)

Data are mean (SD) unless otherwise stated. χ^2^ =  chi-square test; F =  one-way ANOVA; K =  Kruskal-Wallis test; t =  T test; U =  Mann-Whitney U test.

a Numbers denote values on a Likert scale where 0 =  no, 1 =  less than 1 year, 2 = 1–3 years, 3 = 4–6 years, 4 = 7–10 years, and 5 =  more than 10 years of training/playing.

b Numbers denote values on a Likert scale with a range 0 (does never) to 5 (does daily).

c Classification based on BDAE (Goodglass & Kaplan, 1983) Aphasia Severity Rating Scale: scores 0–4  =  aphasia, score 5 =  no aphasia.

d Classification based on the Lateralized Inattention Index of the Balloons Test (Edgeworth, Robertson, & McMillan, 1998).

e Maximum lesion diameter in cm.

### Magnetoencephalography (MEG) measurements

MEG was recorded in a magnetically shielded room (Euroshield Ltd., Finland) at the BioMag laboratory with a 306-channel whole-head magnetometer (Elekta Neuromag Oy, Helsinki, Finland). The position of the subject's head relative to the sensors was determined by measuring the magnetic field produced by four marker coils attached to the scalp [54]. The locations of the coils in relation to cardinal points on the head were determined with a 3D digitizer (Polhemus™, USA). During the measurements, the subjects were presented harmonically rich tones that were delivered binaurally through plastic tubes and earplugs at the intensity of approximately 80 dB sound pressure level (SPL) with a fixed 300 ms stimulus onset asynchrony (BrainStim software). Before each measurement, the audibility of the sounds in both ears was verified by asking the patients if they heard stimuli and if the volume was in a comfortable level. None of the patients reported any trouble in hearing the sounds. For 3 out of 53 patients, the sound volume was lowered to approximately 70 dB SPL since they felt it was too loud. The volume was kept fixed during all 3 measurements.

The stimulus sequence consisted of standard tones (*p* = 0.8; 500, 1000 and 1500 Hz frequency components; 75 ms duration with 5 ms rise and fall times) and deviant tones. The deviant tones had either higher frequency (*p* = 0.1; 575, 1150 and 1725 Hz frequency components) or shorter duration (*p* = 0.1; 25 ms duration) than the standard tones. These stimulus characteristics were selected on the basis of previous MMN and MMNm studies [39], [40], [42], [55]. The tones were presented in random order, except that each deviant tone was preceded by at least two standard tones. In order to control for exogenous effects on the MMN, also two control blocks (referred to hereafter as control-standards) were included [26]. In those, only the higher frequency and the shorter duration tones, which served as deviants in the oddball blocks, were presented at 100% probability. The subjects were instructed to ignore the sound stimuli and focus on watching a silent DVD without subtitles.

Online averaging of the MEG epochs (sampling rate 602 Hz, bandpass filtering 0.1–95 Hz) for the standard and deviant stimuli started 150 ms before and ended 350 ms after stimulus presentation. Epochs with MEG or electro-oculogram (EOG; recorded with electrodes placed above and below the left eye and lateral to the eyes) deflections exceeding 3000 fT/cm or 150 µV, respectively, were discarded from averaging. Recording was continued until approximately 100 accepted artefact-free trials for each deviant type were collected, which took about 10–15 minutes.

For data visualization, the averaged responses to the standard and deviant tones were first digitally filtered (bandpass 1–20 Hz) and baseline-corrected (time interval −50–0 ms before stimulus onset), and then, in order to adjust for head position variability between the measurement sessions, spatially corrected using the MaxFilter™ software (Elekta Neuromag, Finland). MMNm responses to changes in frequency and duration (referred to hereafter as frequency MMNm and duration MMNm, respectively) were determined by subtracting the averaged response to the control-standard tones from the averaged responses to the deviant tones [26]. Source modelling of the MMNm responses was performed from the subtraction curves by using the Minimum Current Estimation (MCE) method (Elekta Neuromag, Finland), which is based on minimum L1-norm estimates and can represent several local or distributed sources [56]. The MCEs were calculated separately for each individual subject at each measurement session (1 week, 3 months, and 6 months post-stroke). Averaged responses were first pre-processed by filtering with a 20 Hz low-pass digital filter and applying a prestimulus baseline (50 ms before stimulus onset) and a detrend baseline (300–350 ms from the stimulus onset) in order to eliminate the effects of measurement noise. A spherical head model was used in calculating MCE solutions, which were then projected onto an averaged brain surface. The origin of this model was determined individually for each subject on the basis of a 3D set of T1-weighted anatomical MRIs by fitting a sphere to the curvature of the outer surface of the brain.

**Figure 2:**
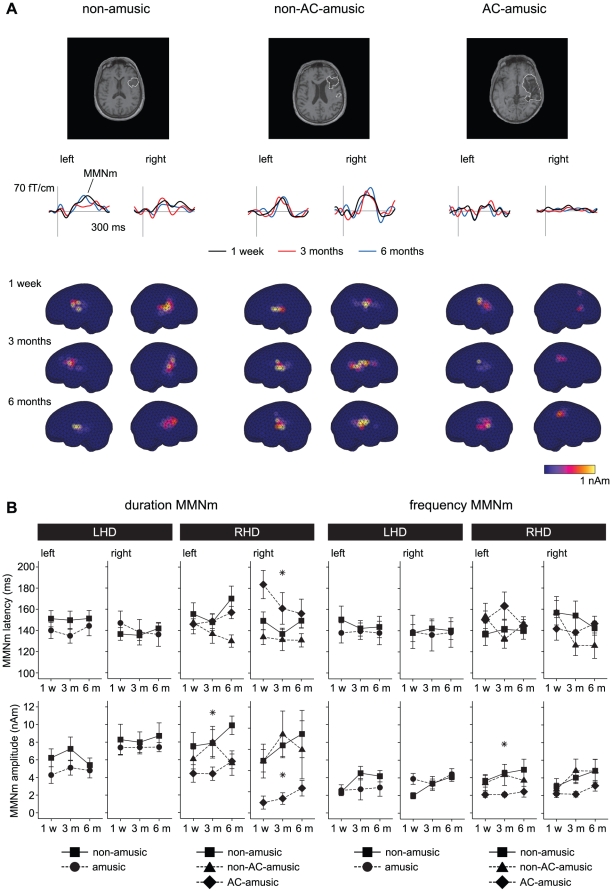
MMNm in amusic and non-amusic patients at different stages of stroke recovery. (A) Case examples illustrating the typical recovery of the duration MMNm in non-amusic, non-AC-amusic and AC-amusic patients with right hemisphere damage. The MRI images (upper) show the location of the lesion. Changes in the strength of the MMNm in the left and right hemispheres are shown with subtraction curves from individual MEG channels over the temporal lobes (middle) and with source modelling performed using the MCE method (lower). (B) Group results of the latency and amplitude of the duration MMNm and the frequency MMNm in the left and right hemispheres 1 week (1 w), 3 months (3 m), and 6 months (6 m) post-stroke. Data (mean ± SEM) are shown for non-amusic (n = 12) and amusic (n = 12) patients with left hemisphere damage (LHD) and for non-amusic (n = 9), non-AC-amusic (n = 9), and AC-amusic (n = 11) patients with right hemisphere damage (RHD). *p<0.05 in mixed-model ANOVA (Group effect).

After calculating the MCE, we identified the source of the MMNm in each hemisphere by selecting a region of interest (ROI) that produced the strongest response that was within the time window of 100–300 ms from tone onset and followed the vertical (“downward”) dipolar orientation typical of the MMNm [57]. Using graphical interface of the Neuromag MCE software, the ROI was selected individually for each patient at each measurement sessions so that it always produced the highest amplitude response within the hemisphere (for case examples illustrating the recovery-related change in the MMNm derived from the MCE analysis, see Figure 2 and [42]). In line with the literature on the typical sources of the MMN in the normal brain [57]–[62], the ROIs were primarily located in the temporal lobe, extending in some cases also frontally or parietally. MMNm latency was determined from the peak of the response. MMNm amplitude was determined as the mean amplitude within a 50-ms time window centred at the peak of the response.

### Statistical analysis

Differences between the amusic and non-amusic patients in demographical, musical, and clinical characteristics were analyzed with chi-square (likelihood ratio) tests, t-tests, Mann-Whitney U tests, univariate ANOVAs, and Kruskal-Wallis tests. Group differences in MMNm parameters, MBEA scores and neuropsychological test scores at different stages of stroke recovery were analyzed using univariate and mixed-model ANOVAs. The Greenhouse-Geisser epsilon was used to correct for sphericity. The relationships between the MMNm responses and the MBEA scores were analyzed with correlation analyses (Pearson, 2-tailed). The level of statistical significance was set at *p*<0.05. All statistical analyses were performed using SPSS (version 15.0). Missing values in test scores were considered missing at random.

## Results

In order to determine whether amusic and non-amusic patients with left hemisphere damage (LHD) and right hemisphere damage (RHD) would differ in their MBEA and MMNm recovery profiles, we first divided the large patient sample (n = 53) into LHD patients (n = 24) and RHD patients (n = 29). Furthermore, in order to determine the impact of auditory cortex (AC) damage on amusia, the amusic group was further divided to patients who had a lesion extending to the AC and patients whose lesion spared the AC (referred to hereafter as the “AC-amusic” and “non-AC-amusic” patients, respectively). Because 11 out of 14 AC-amusic patients had RHD, this division was done only in the RHD subgroup. Thus, all analyses were done separately comparing amusic (n = 12) and non-amusic (n = 12) patients within the LHD subgroup and AC-amusic (n = 11), non-AC-amusic (n = 9) and non-amusic (n = 9) patients within the RHD subgroup. This provided a balanced number of patients for performing group comparisons and also enabled us to compare the MMNm in the left and right hemispheres.

Before performing group comparisons, we analyzed whether there were differences in relevant demographical or clinical variables between the amusic and the non-amusic patients (Table 2). Within the LHD subgroup, the amusic patients had less education [*t*(22)  = 3.78, *p* = 0.002] and larger lesions [*t*(22)  = −2.85, *p* = 0.009] and were also slightly older [*t*(22) = −2.07, *p* = 0.051] than the non-amusic patients. Within the RHD subgroup, there was a marginally significant group difference in lesion size [*F*(2, 26) = 2.68, *p* = 0.087]. Post hoc testing (LSD) indicated that the lesions were larger in the AC-amusic group than in the non-amusic group (*p* = 0.03) but did not differ between the AC-amusic and non-AC-amusic groups (*p* = 0.197). In order to account for these differences, we included education, age, and lesion size as covariates in all analysis of LHD patients and lesion size as a covariate in all analysis of RHD patients. Notably, there were no differences in lesion location between the amusic and the non-amusic LHD patients. Due to the rarity of naturally occurring pure AC lesions, the lesions of the AC-amusic RHD patients extended more often to the temporal lobe than the lesions of the non-amusic RHD patients (*χ^2^* = 7.65, *p* = 0.006) and to the parietal lobe and the insula more often than the lesions of the non-amusic RHD patients (*χ^2^* = 7.74, *p* = 0.005 and *χ^2^* = 7.65, *p* = 0.006, respectively) or the non-AC-amusic RHD patients (*χ^2^* = 7.74, *p* = 0.005 and *χ^2^* = 5.45, *p* = 0.02, respectively). However, since the perception and discrimination of basic acoustic features, such as frequency and duration, involves primarily temporal and frontal areas [2], the differences in parietal and insular lesion extent were not considered important in comparing the groups. The AC-amusic and the non-AC-amusic RHD patients did not differ in the proportion of frontal (100% in both) or temporal lobe lesions (100% vs. 89%, *χ^2^* = 1.66, *p* = 0.197), suggesting that any differences between them could be attributed to the AC damage. Finally, we also compared the amusic LHD patients (n = 12) and the amusic RHD patients (n = 20) in demographical and clinical variables. Compared with the amusic LHD group, the amusic RHD group had a higher proportion of women (*χ^2^* = 4.15, *p* = 0.042) and their lesions extended more often to frontal (*χ^2^* = 4.15, *p* = 0.042), temporal (*χ^2^* = 6.64, *p* = 0.01), insular (*χ^2^* = 6.54, *p* = 0.011), and subcortical (*χ^2^* = 4.97, *p* = 0.026) areas. However, their overall lesion sizes did not differ [*t*(22)  = −0.84, *p* = 0.408]. Thus, female gender was included as a covariate when comparing these groups.

### MBEA performance in amusic and non-amusic patients

Group differences in the MBEA average score (Figure 1) were analyzed first using univariate ANOVAs for the 1-week post-stroke data and then, in order determine whether the group differences were stable over time, also using mixed-model ANOVAs for the 1-week and 3-month post-stroke data. Separate analyses were performed for the LHD (amusic vs. non-amusic) and RHD (AC-amusic vs. non-AC-amusic vs. non-amusic) subgroups. In the LHD subgroup, the MBEA average score was significantly lower in the amusic than in the non-amusic patients at the 1-week post-stroke stage [*F*(1, 19)  = 17.8, *p* = 0.0005] and also throughout the 3-month post-stroke period [*F*(1, 19)  = 17.82, *p* = 0.0005]. Also in the RHD subgroup, there were significant group differences on the MBEA average score both 1 week post-stroke [*F*(2, 25) = 40.36, *p*<0.0001] and during the 3-month post-stroke period [*F*(2, 25) = 25.13, *p*<0.0001]. Post hoc testing (LSD) showed that the MBEA average score was clearly lower in both the AC-amusic and the non-AC-amusic groups than in the non-amusic group (*p*<0.0001 in both comparisons) but also lower in the AC-amusic group than in the non-AC-amusic group (p<0.05) during the 3-month follow-up. Using the same amusia classification criterion as in the 1-week post-stroke stage, a significantly higher percentage of the AC-amusic patients than the non-AC-amusic patients also remained amusic at the 3-month post-stroke stage (91% vs. 44%, *χ^2^* = 5.37, *p* = 0.021). Finally, we also compared the amusic LHD patients (n = 12) and the amusic RHD patients (n = 20) and found that the MBEA average score was lower in the amusic RHD patients at the 1-week stage [*F*(1, 29) = 8.29, *p* = 0.007] and throughout the 3-month period [*F*(1, 29)  = 5.58, *p* = 0.025]. However, similar improvement in the MBEA average score from the 1-week to the 3-month stage was seen in both amusic LHD and RHD patients.

In summary, these results suggest that RHD causes a more severe deficit in music perception than LHD but the laterality of cerebral damage does not influence the behavioural recovery of amusia. Moreover, at least in right hemisphere stroke, damage to the auditory cortex seems to be a crucial factor limiting the recovery of amusia.

### MMNm in amusic and non-amusic patients

At the 1-week post-stroke stage, both frequency and duration deviants elicited MMNm responses that peaked around 150 ms. The MMNn mean amplitudes (Table 3) differed significantly from zero in both ipsilesional and contralesional hemispheres in all patient groups. Group differences in the latency and amplitude of the duration MMNm and the frequency MMNm responses (Figure 2) were analyzed using univariate ANOVAs for the 1-week post-stroke data and mixed-model ANOVAs for the 1-week, 3-month, and 6-month post-stroke data. In the LHD subgroup, no significant differences between the amusic and the non-amusic patients were observed at the 1-week post-stroke stage or during the 6-month period. In the RHD subgroup, there were significant group differences in right hemisphere duration MMNm latency [*F*(2, 25) = 4.47, *p* = 0.022] and amplitude [*F*(2, 25) = 3.56, *p* = 0.043] 1 week post-stroke with post hoc tests (LSD) showing a longer latency and a smaller amplitude in the AC-amusic group than in the non-amusic (*p* = 0.029 and 0.017) or non-AC-amusic (*p* = 0.002 and 0.017) groups. These Group effects remained significant also during the 6-month period [*F*(2, 25) = 3.5, *p* = 0.046 and *F*(2, 25) = 3.47, *p* = 0.047]. Additionally, significant Group effects were also observed in the amplitude of the duration MMNm [*F*(2, 25) = 4.55, *p* = 0.021] and the frequency MMNm [*F*(2, 25) = 3.84, *p* = 0.035] in the left hemisphere during the 6-month period. Post hoc tests showed that the left hemisphere duration MMNm amplitude was smaller in the AC-amusic group than in the non-amusic group (*p* = 0.006) and the left hemisphere frequency MMNm amplitude was smaller in the AC-amusic group than in both non-amusic (*p* = 0.014) and non-AC-amusic (*p* = 0.056) groups. Importantly, there were no significant differences between the non-AC-amusic and non-amusic groups during the 6-month follow-up (p = 0.132–0.937).

**Table 3:** MMNm amplitudes 1 week post-stroke.

Lesion	Group	Hemisphere	Deviant	Mean^a^	SD	t value^b^	P value
LHD	non-amusic	left	duration	6.2	3.5	6.12	<0.0001
		right	duration	8.3	6.0	4.75	0.0005
		left	frequency	2.4	1.2	6.86	<0.0001
		right	frequency	2.0	1.2	5.63	0.0001
	amusic	left	duration	4.5	3.1	5.04	0.0003
		right	duration	7.7	2.8	4.32	<0.0001
		left	frequency	2.5	2.0	9.74	0.001
		right	frequency	3.8	2.0	6.67	<0.0001
RHD	non-amusic	left	duration	7.5	4.6	5.13	0.001
		right	duration	6.9	3.2	6.72	0.0002
		left	frequency	3.6	2.1	4.82	0.0008
		right	frequency	3.0	2.6	4.47	0.008
	non-AC-amusic	left	duration	6.1	3.4	3.77	0.0007
		right	duration	6.9	4.8	4.0	0.003
		left	frequency	3.4	2.6	4.32	0.004
		right	frequency	2.7	2.1	5.39	0.005
	AC-amusic	left	duration	4.4	3.3	3.46	0.001
		right	duration	3.0	2.0	5.13	0.0006
		left	frequency	2.1	1.0	6.55	<0.0001
		right	frequency	2.2	1.4	4.91	0.0004

LHD  =  left hemisphere damage, RHD  =  right hemisphere damage.

a MMNm mean amplitude (nAm).

b One-sample T test (against zero).

**Figure 3:**
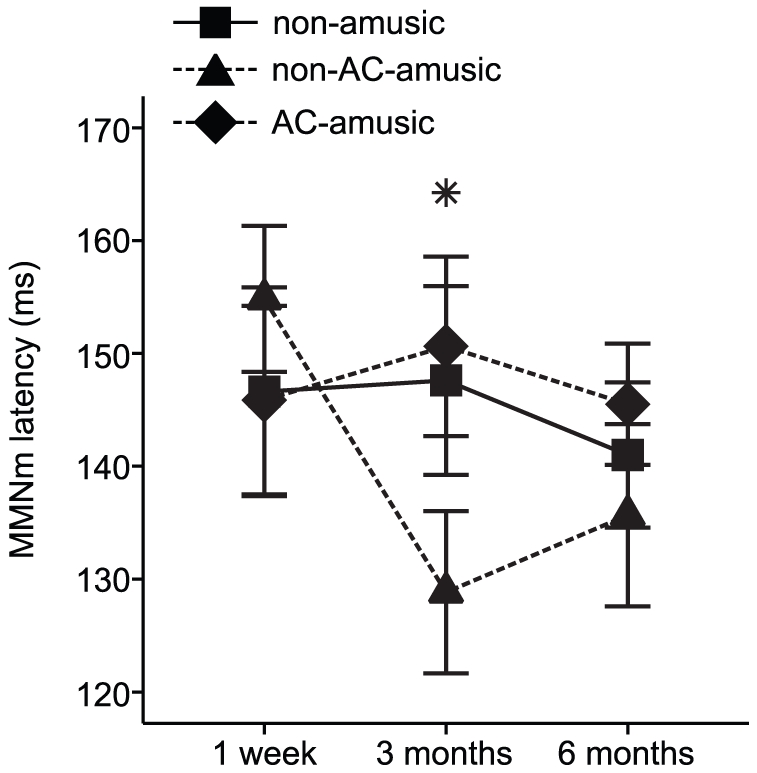
Latency of the averaged frequency MMNm response. Data (mean ± SEM) are shown for non-amusic (n = 9), non-AC-amusic (n = 9), and AC-amusic (n = 11) patients with right hemisphere damage. *p<0.05 in mixed-model ANOVA (Time x Group interaction).

The mixed-model ANOVA also yielded a significant multivariate Time x Group interaction for the frequency MMNm latency [Wilks' λ = 0.68, *F*(8, 98) = 2.59, *p* = 0.013]. Post hoc testing (LSD) was performed on the change scores (3 months minus 1 week, 6 months minus 1 week) of the average of the left and right hemisphere frequency MMNm latency (Figure 3) and showed that the overall frequency MMNm latency decreased more in the non-AC-amusic patients than in the AC-amusic patients (*p* = 0.011) or the non-amusic (*p* = 0.031) patients during the first 3 months of recovery.

**Figure 4:**
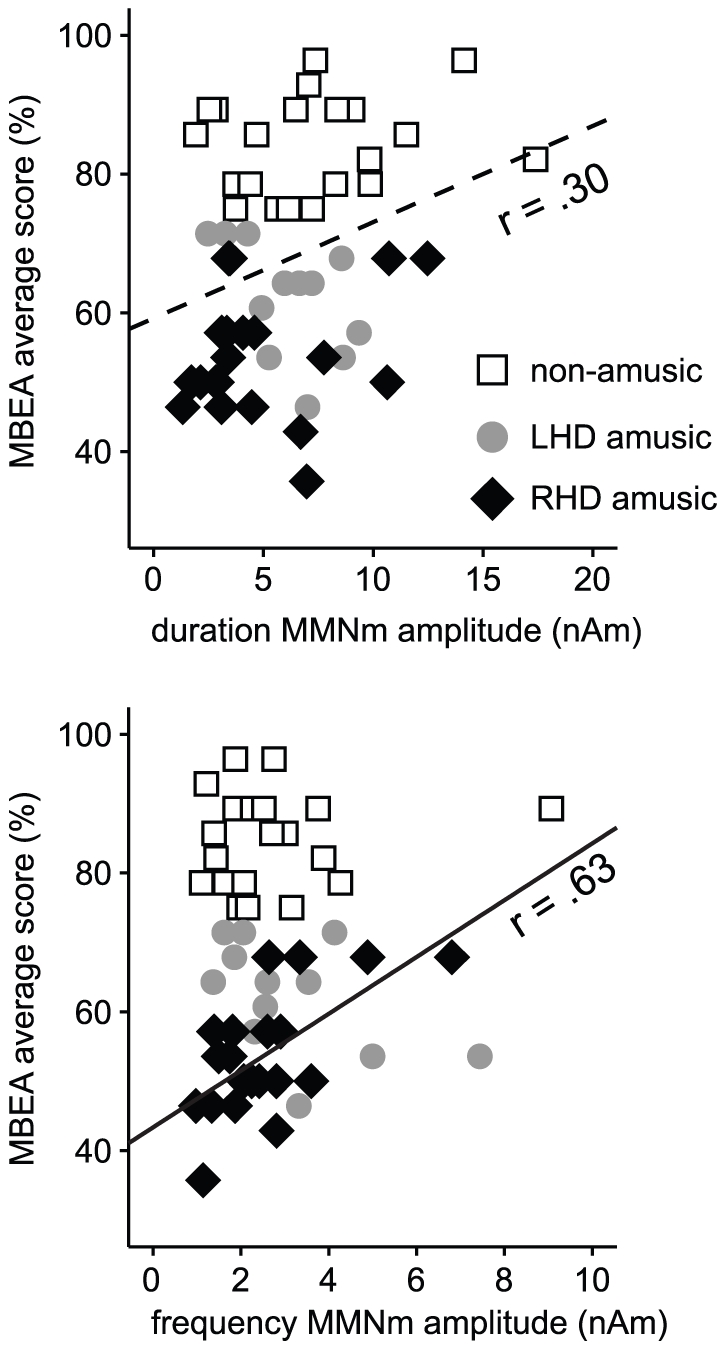
Relationship between MBEA scores and MMNm responses 1 week after the stroke. Scatterplots indicating the correlation between the MBEA average score and the duration and frequency MMNm overall amplitudes (average of left and right hemisphere responses) are shown for non-amusic patients (n = 21), amusic patients with left hemisphere damage (LHD amusic, n = 12) and amusic patients with right hemisphere damage (RHD amusic, n = 20). Regression lines are shown only for statistically significant correlations. The dashed line in the upper figure is for all patients (n = 53) and the solid line in the lower figure is for RHD amusic patients.

In order to determine if deficient auditory discrimination was related to poor music perception, we performed correlation analyses (Pearson, 2-tailed) between the MMNm responses and the MBEA average score at the 1-week post-stroke stage (Figure 4). For the MMNm, the averages of the left and right hemisphere response amplitudes were used. Across all patients, there was a small but significant correlation between the MBEA score and the duration MMNm amplitude (*r* = .30, *p* = 0.031). In contrast, a larger correlation was observed in the amusic RHD patients between the MBEA score and the frequency MMNm amplitude (*r* = .63, *p* = 0.003). In the amusic LHD patients, there were no positive correlations between MBEA performance and MMNm.

In summary, poor performance on the MBEA test was related to weaker frequency MMNm responses in RHD amusic patients but not in LHD amusic patients. Within the RHD subgroup, clearly diminished MMNm responses were observed especially in the AC-amusic patients whereas the non-AC-amusic patients did not differ from the non-amusic patients. This suggests that acquired amusia may not always involve a deficit in auditory discrimination.

### Cognitive performance in amusic and non-amusic RHD patients

**Figure 5:**
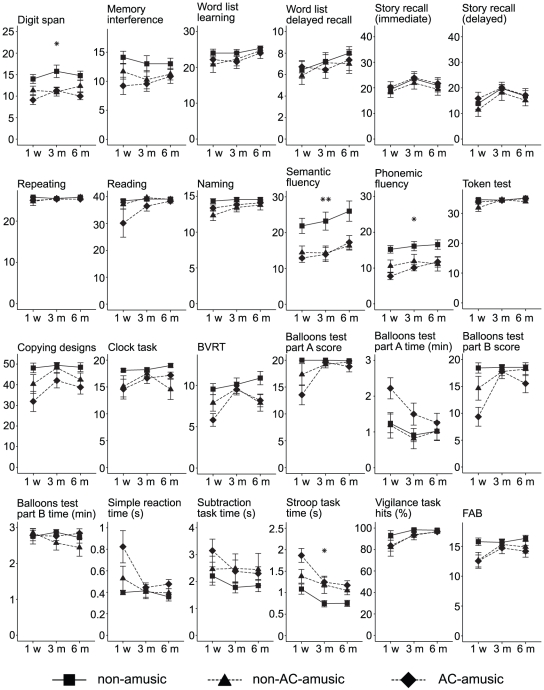
Cognitive performance of right hemisphere-damaged amusic and non-amusic patients at different stages of stroke recovery. Data (mean ± SEM) are shown for right hemisphere-damaged patients with no amusia (n = 9), amusia without auditory cortex damage (non-AC-amusic, n = 9), and amusia with auditory cortex damage (AC-amusic, n = 11) 1 week (1 w), 3 months (3 m), and 6 months (6 m) post-stroke. The Y axis is scaled to the maximum score (except in fluency and reaction time tests). BVRT  =  Benton Visual Retention Test, FAB  =  Frontal Assessment Battery (see Table 1 for test descriptions). *p<0.05 and **p<0.01 in mixed-model ANOVA (Group effect).

Previously, we found that amusic patients performed worse than non-amusic patients especially on tests of attention, working memory and executive functioning throughout the 6-month post-stroke period [38]. In order to determine if this effect depended on the location of the lesion, group differences on the neuropsychological tests (Figure 5) were analyzed in RHD patients using univariate ANOVAs for the 1-week post-stroke data and mixed-model ANOVAs for the 1-week, 3-month, and 6-month post-stroke data. At the 1-week post-stroke stage, significant group differences were observed for performance on the phonemic [*F*(2, 25) = 6.13, *p* = 0.007] and semantic [*F*(2, 25) = 4.97, *p* = 0.015] fluency tasks and for the reaction times on the Stroop task [*F*(2, 22) = 5.21, *p* = 0.014]. These group effects remained significant also throughout the 6 month post-stroke period [*F*(2, 25) = 3.54, *p* = 0.044; *F*(2, 25) = 6.3, *p* = 0.006; and *F*(2, 22) = 4.05, *p* = 0.032]. Additionally, the mixed-model ANOVA yielded a significant Group effect also for the digit span test [*F*(2, 23) = 4.21, *p* = 0.028]. Post hoc testing (LSD) indicated that both the AC-amusic patients and the non-AC-amusic patients performed significantly worse than the non-amusic patients on the digit span (*p* = 0.012 and 0.033), phonemic fluency (*p* = 0.023 and 0.035), and semantic fluency (*p* = 0.008 and 0.003) tests throughout the 6-month period. Compared with the non-amusic patients, the reaction times on the Stroop task were slower in the AC-amusic patients (*p* = 0.011) and, to a lesser degree, also in the non-AC-amusic patients (*p* = 0.066). A significant group difference was observed also for performance on the Balloons test part B at the 1 week post-stroke stage [*F*(2, 25) = 3.59, *p* = 0.042] but this effect did not remain significant at later stages. Importantly, there were no significant differences between the AC-amusic patients and the non-AC-amusic patients in any test (*p* = 0.313–0.792).

In summary, both the AC-amusic patients and the non-AC-amusic patients performed worse than the non-amusic patients specifically on tests of working memory (digit span), attention (Stroop), and cognitive flexibility (fluency tasks) during the 6-month follow-up. Together with the MMNm results, this suggests that acquired amusia, which does not result from damage to the AC and, thus, does not involve a deficit in auditory discrimination, can be considered more as a by-product of domain-general cognitive deficits mediated primarily by frontal lobe dysfunction.

## Discussion

The results of the present study reveal that deficits in both basic auditory encoding and in higher domain-general cognitive processing underlie acquired amusia after MCA stroke, and, furthermore, that their relative contribution to amusia depends on the location of the cerebral damage. First of all, we found that amusia caused by right hemisphere damage (RHD), especially in temporal and frontal brain areas, was more severe than the amusia caused by left hemisphere damage (LHD). Moreover, correlation analyses indicated that smaller duration MMNm amplitudes were associated with lower MBEA scores across patients whereas smaller frequency MMNm amplitudes correlated with lower MBEA scores only in amusic RHD patients. In contrast, the amusic and the non-amusic LHD patients did not differ in their MMNm responses and there was also no correlation between MMNm and MBEA scores in the amusic LHD patients. These results suggest that the deficit in music perception may be related to deficient pitch discrimination in the brain only in amusia caused by RHD. Previously, clinical studies have shown that a deficit in perceiving pitch within melodies is more typically caused by RHD than LHD [63]–[66]. Similarly, evidence from neuroimaging studies of healthy subjects suggests that comparing the pitch of two notes within melodies, like in the MBEA, activates a network of frontal and temporal areas in the right hemisphere (e.g. [67]).

Evidence from previous clinical and neuroimaging studies strongly indicates that right auditory cortical (AC) structures play an important role in musical pitch processing. For example, damage to the right AC has been shown to produce a severe deficit in discriminating melodies [65], [68] and perceiving pitch [64], [69], [70], [71] as well as in producing pitch when singing [71]. Correspondingly, neuroimaging studies of healthy subjects have shown that the right AC and other auditory areas in the right hemisphere are active during passive listening of melodies [34], [67], [72] and respond especially to small pitch changes [73]–[76]. However, when the subject has to perform an active task (e.g., same-different discrimination of two melodies or of two pitches within a melody), a network of areas in the frontal lobe, including the precentral, the inferior frontal, and the dorsolateral prefrontal cortical areas as well as the anterior cingulate cortex, becomes activated in addition to the temporal areas [34], [67], [77], [78]. Based on these results, Brown and Martinez [34] have argued that discrimination processing involves both domain-specific sensorimotor areas and domain-general areas involved in working memory and error detection.

In principle, this evidence from previous studies suggests that the music perception deficit in the amusic brain, as indicated by poor performance on the MBEA, could be caused by different mechanisms depending on which part of the music processing network is damaged. Thus, damage to the AC or other temporal areas could cause a deficit in the analyses of the basic acoustical features, such as pitch, timbre and duration, which are the fundamental elements of music. In contrast, damage to frontal areas could cause a deficit in the more cognitive, conscious analysis of the music information where domain-general cognitive functions, such as attention and working memory, also come into play. In line with this idea, results from previous stroke patient studies have shown that amusic patients have relatively general deficits in neural sound processing and attention orienting, including reduced MMN responses to pitch changes [36] and decreased P3a responses to environmental sounds [37] as well as deficient performance on a behavioural auditory alertness test [37]. However, the small number of patients (n = 12) in these studies precluded any conclusions about the effect of lesion location on these deficits. Our results showed that the AC-amusic patients with extensive RHD involving the AC and other temporal lobe areas as well as the frontal lobe had smaller and slower MMNm responses than the non-amusic RHD patients or the non-AC-amusic RHD patients whose lesions included frontal and temporal areas but spared the AC. In addition, both the AC-amusic patients and the non-AC-amusic patients performed worse than the non-amusic patients on the digit span, verbal fluency, and Stroop tasks, indicating a more severe deficit in working memory, cognitive flexibility, and attention. Taken together, these results suggest that domain-general cognitive deficits may be the primary mechanism underlying amusia without AC damage whereas amusia caused by a large lesion extending to frontal and temporal areas and including the AC is associated with both basic auditory and cognitive deficits.

Regarding the recovery of amusia, very little is currently known. Previously, we found that improvement on the MBEA correlated mostly with the recovery of focused attention, verbal memory, and visuospatial perception and attention in amusic patients from the acute to the 3-month post-stroke stage [38]. In the present study, we observed that the amusia caused by RHD without AC damage was less severe than the amusia caused by RHD with AC damage during the 3-month post-stroke period. Overall, less than half (44%) of the non-AC-amusic RHD patients could still be classified as amusic (scoring below the 75% cut-off in the MBEA Scale and Rhythm subtests) at the 3 month stage whereas a vast majority (91%) of the AC-amusic RHD patients remained amusic at the 3 month stage. Thus, severe and persistent amusia seems to be caused especially by extensive damage to the right hemisphere covering the AC and other temporal and frontal lobe areas. Interestingly, the non-AC-amusic RHD patients also showed faster pitch discrimination, as indicated by the shortening of the frequency MMNm latency during first the 3 months of recovery, than the AC-amusic RHD patients or the non-amusic RHD patients. Since the severity of cognitive deficits was comparable between the non-AC-amusic and the AC-amusic patients throughout the follow-up, it is plausible that the speed-up in pitch discrimination taking place in the early post-stroke stage may be one important mechanism underlying the recovery of music perception in the non-AC-amusic patients.

There are a few notable methodological limitations to this study, which should be taken into account when interpreting the results. Firstly, due to time constraints, we were not able to include audiometry to determine the basic auditory and hearing capabilities of the patients in the study. However, since we (1) excluded all patients with a history of problems in basic auditory perception, (2) did not observe any notable hearing deficits in the patients during normal conversation or during the neuropsychological assessment, (3) made sure that the auditory stimuli MBEA as well as those used in the MEG were clearly audible to the patients, and (4) verified that the amplitude of the MMNm differed from zero in all the patient groups (including the AC-amusic RHD patients) at the 1-week post-stroke stage, we can be confident that the MBEA or MMNm results were not biased by potential problems in basic auditory perception. Secondly, the small number of amusic patients with left AC damage (n = 3) limits the conclusions about the role of AC damage in amusia only to RHD patients. Due to the fact that focal damage restricted to the AC is extremely rare after an ischemic stroke, patients with left AC damage often have large lesions extending to temporal, frontal, parietal, and subcortical areas, and, consequently, are severely aphasic, which naturally precludes their recruitment. Thus, studies with larger sample sizes of LHD patients (especially with AC damage) are still needed to provide information about the potential neural and cognitive correlates of amusia after LHD and also to gain more insight about the relationship between aphasia and amusia. Thirdly, the large overlap in the lesion locations (i.e. the lesions typically covered many cortical and subcortical areas) of our patient sample and, consequently, the relatively rough anatomical classification used in the present study precludes making more precise inferences about the roles of different brain structures in amusia. In the future, studies that use more advanced analyses of lesion locations (for example with voxel-based morphometry) on a larger patient sample or that focus on patients who have specific damage to certain key brain structures (e.g., AC and other temporal areas, IFG) would help to shed more light on how different areas contribute to music perception in the brain.

In conclusion, the present data indicate that the severity and recovery of amusia caused by cerebral damage as well as the relative contribution of perceptual and cognitive factors in amusia depend on which parts of the large-scale neural network governing music perception and cognition are damaged. It seems that, at least in the right hemisphere, damage to the AC together with damage to other temporal and frontal structures leads to a severe and persistent form of amusia that is characterized by deficits in both low-level auditory processing and higher cognitive functions. In contrast, damage to temporal and frontal areas that spares the AC results in a more transient form of amusia, which is related primarily to cognitive deficits. Clinically, this information may be important in helping derive a more accurate prognosis of the musical deficit. Identifying whether the amusic patient might benefit more from the training of musical perceptual skills (e.g., melody discrimination training [79]) or cognitive skills (e.g., attention and memory training) would also be important in guiding the development and application of potential rehabilitation interventions for amusia.
